# Quarterly persistence as a signal prioritization framework in FAERS: an analysis of the 2025 public-use releases

**DOI:** 10.1007/s00210-026-05505-4

**Published:** 2026-06-08

**Authors:** Juan Alberto Martínez-Cadena, Violeta Garza-Villafuerte

**Affiliations:** 1 Departamento de Matemáticas, Universidad Autónoma Metropolitana-Iztapalapa, CDMX, Iztapalapa, 09340 México; 2 Departamento de Física, Universidad Autónoma Metropolitana-Iztapalapa, CDMX, Iztapalapa, 09340 México

**Keywords:** FAERS, Pharmacovigilance, Disproportionality analysis, Temporal persistence, Signal prioritization, Sensitivity analysis

## Abstract

To develop and apply a quarterly pharmacovigilance workflow for the 2025 FDA Adverse Event Reporting System (FAERS) public-use XML releases, with emphasis on temporal persistence, cohort sensitivity, and prioritization-oriented signal characterization. The raw input consisted of 12 quarterly FAERS XML files (approximately 8.58 GB), which were parsed into report-level drug-reaction pairs using a chunked workflow. The main analysis used a PS-clinical cohort including primary suspect drugs from serious reports, whereas a sensitivity analysis expanded this definition to a PS + SS-clinical cohort by including secondary suspect drugs. Quarterly product-reaction pairs were screened using a disproportionality rule requiring ${n}_{11}\ge 3$, proportional reporting ratio $\mathrm{P}\mathrm{R}\mathrm{R}\ge 2$, ${\chi }^{2}\ge 4$, and a lower 95% confidence bound of the reporting odds ratio greater than 1. A more conservative subset was defined by ${n}_{11}\ge 10$, followed by post-screening refinement for temporal comparison. Under the PS-clinical cohort, the quarterly retained sets ranged from 41,897 to 47,220 pairs, and the annual union contained 82,565 unique retained pairs. Of these, 18,629 (22.56%) were detected in all four quarters. Pairwise Jaccard similarities ranged from 0.4027 to 0.4845, indicating moderate but structured temporal continuity, with the strongest overlap between Q3 and Q4. Fully persistent 4/4 pairs showed higher support counts and larger mean ${\chi }^{2}$ values than quarter-specific 1/4 pairs, whereas mean PRR did not differ materially between these groups. Within the 4/4 core, most pairs were relatively stable over time, while only a small subset showed marked temporal imbalance. Expansion to the PS + SS-clinical cohort substantially increased both the retained signal space and the 4/4 core, while leaving the overall temporal overlap pattern broadly similar. Quarterly persistence may provide an additional and interpretable dimension for internal FAERS-based signal characterization and prioritization. Rather than replacing disproportionality metrics, it may complement them by highlighting reproducibility, continuity, and temporal structure across quarterly retained signal sets. External validation against independent pharmacovigilance reference standards remains an important direction for future work.

**Supplementary Information:**

The online version contains supplementary material available at 10.1007/s00210-026-05505-4.

## Introduction

Spontaneous reporting systems play a central role in post-marketing pharmacovigilance by enabling large-scale signal detection under real-world conditions, often before more formal epidemiological evidence becomes available (Bate and Evans 2009; Harpaz et al. 2012; Fusaroli et al. 2024). Among these systems, the U.S. Food and Drug Administration Adverse Event Reporting System (FAERS) is particularly important because of its size, public accessibility, and widespread use in signal detection practice (U.S. Food and Drug Administration 2025; Huang et al. 2014). Within this setting, disproportionality methods such as the proportional reporting ratio (PRR), reporting odds ratio (ROR), chi-square statistics, information component (IC), and empirical Bayesian approaches have become standard tools for identifying product-reaction pairs reported more frequently than expected relative to background reporting patterns (Bate et al. 1998; Bate and Evans 2009; Candore et al. 2015; Evans et al. 2001; Cutroneo et al. 2024; van Puijenbroek et al. 2002).

At the same time, signals derived from spontaneous reporting systems require cautious interpretation. FAERS data are affected by well-known limitations, including under-reporting, stimulated reporting, heterogeneous data quality, duplicate submissions, changes in product utilization, and the absence of exposure denominators (Cutroneo et al. 2024; Fusaroli et al. 2024; Harpaz et al. 2012). For this reason, disproportionality analyses are more appropriately viewed as signal-generating tools than as instruments for causal inference (Cutroneo et al. 2024; Evans et al. 2001; van Puijenbroek et al. 2002). Nevertheless, they remain highly valuable for prioritization, especially when embedded within transparent analytical workflows, sensitivity analyses, and clinically interpretable post-screening strategies (Candore et al. 2015; Jiao et al. 2024; Khouri et al. 2021).

In practice, disproportionality studies are commonly presented as cross-sectional snapshots, even though FAERS is updated on a quarterly basis (U.S. Food and Drug Administration 2025; Huang et al. 2014). This quarterly structure offers an opportunity to examine signal detectability over time rather than within a single isolated release. A product-reaction pair retained in only one quarter may reflect a transient pattern, whereas repeated detection across multiple quarters may indicate a more reproducible reporting signal deserving closer attention. Although the pharmacovigilance literature provides substantial guidance on signal detection methodology and on the strengths and limitations of spontaneous reporting systems, less emphasis has been placed on persistence across quarterly releases, pairwise overlap between retained quarterly signal sets, and repeated detectability over successive public data updates.

A temporal perspective is also relevant because the retained signal space in each quarter depends on several analytical decisions, including XML parsing strategy, duplicate handling, drug role selection, seriousness filtering, disproportionality thresholds, and post-screening refinement. Comparative methodological studies have shown that signal detection results may vary across algorithms, databases, and analytical definitions (Candore et al. 2015; Khouri et al. 2021; Coste et al. 2023; Vogel et al. 2020). In this context, differences between analytical settings should not necessarily be interpreted as mere disagreement; they may also provide information about robustness and reproducibility (Khouri et al. 2021). Accordingly, a quarterly pharmacovigilance workflow should not only rank signals within a single release, but also characterize temporal overlap, persistence classes, and the sensitivity of conclusions to reasonable changes in cohort definition.

The present study addresses these questions using all 2025 FAERS public-use XML files. Specifically, we sought (i) to implement a memory-efficient workflow that converts raw XML files into report-level drug-reaction pairs, (ii) to define a primary PS-clinical cohort and derive quarterly retained signal sets after statistical screening and post-screening refinement, (iii) to quantify annual persistence through the 1/4, 2/4, 3/4, and 4/4 classes, (iv) to evaluate sensitivity under a broader PS + SS-clinical cohort, and (v) to characterize the persistent 4/4 core in terms of support, temporal variability, and a brief qualitative review.

The aim of this work is not to claim clinical causality but to provide an interpretable framework for temporal signal prioritization. We therefore focus on persistence, overlap, stability, and sensitivity as complementary dimensions of pharmacovigilance assessment. Under this perspective, quarterly retained signal sets may be viewed not merely as isolated disproportionality outputs, but as temporally structured analytical objects. Quarterly persistence, in turn, may offer an additional and practical layer for distinguishing quarter-specific associations, reproducible annual cores, and persistent but unevenly expressed signals within the broader FAERS reporting landscape.

## Methods

### Data source and quarterly raw files

This study used the public-use XML releases of the U.S. Food and Drug Administration Adverse Event Reporting System (FAERS) for the four quarters of 2025, downloaded from the FDA quarterly public data distribution (U.S. Food and Drug Administration 2025). The raw input comprised 12 XML files, three per quarter, with a total size of approximately 8.58 GB. The file sizes were 640.35, 703.81, and 851.00 MB for Q1; 614.68, 659.96, and 803.08 MB for Q2; 772.97, 816.16, and 783.36 MB for Q3; and 658.93, 677.24, and 799.76 MB for Q4. Preliminary inspection confirmed the expected XML *hierarchy*, *including safetyreport*, *patient*, *drug*, and *reaction elements*.

Because the quarterly XML files were too large for convenient single-pass in-memory parsing, the workflow was implemented as a file-wise and block-wise pipeline, with intermediate outputs written directly to disk.

### Pair construction and chunked parsing

Each safety report was converted into report-level drug-reaction pairs by linking drug entries to the reaction preferred terms recorded within the corresponding patient section. Only the variables required for downstream quarterly signal detection were retained: *quarterly source*, *safetyreportid*, *receipt date*, *seriousness indicators*, *drug characterization*, *medicinal product*, and *reaction preferred term*.

Duplicate drug-reaction pairs within the same report were removed using the tuple (*safetyreportid*, *drugcharacterization*, *medicinalproduct*, *reactionmeddrapt*). Parsing was performed in blocks of 2000 safety reports, and each processed block was written immediately to Parquet format. This chunked strategy reduced memory demands and provided an auditable sequence of intermediate parsed files.

### Study cohorts

The main analysis was based on a PS-clinical cohort, defined as report-pair records satisfying both of the following conditions: (i) drugcharacterization = 1, corresponding to primary suspect drugs; and (ii) the report met a seriousness criterion, either through the overall serious flag or through at least one specific seriousness indicator. A broader sensitivity cohort, denoted PS + SS-clinical, included both primary and secondary suspect drugs, that is, drugcharacterization in {1, 2}, while preserving the same seriousness filter.

Under the PS-clinical definition, the quarterly numbers of unique reports were 223,449 in Q1, 220,734 in Q2, 252,805 in Q3, and 216,833 in Q4. After aggregation into distinct product-reaction combinations for quarterly signal detection, the corresponding numbers of evaluated pairs were 717,631, 700,560, 772,205, and 723,982, respectively.

Under the broader PS + SS-clinical definition, the quarterly numbers of unique reports were 223,995 in Q1, 221,407 in Q2, 253,547 in Q3, and 217,566 in Q4. The corresponding numbers of evaluated product-reaction pairs increased to 1,774,011, 1,720,658, 1,815,513, and 1,745,641, respectively. Thus, inclusion of secondary suspect drugs had only a modest effect on the number of retained reports but substantially expanded the quarterly product-reaction signal space.

### Disproportionality screening

For each quarter and cohort definition, the retained report-level pairs were aggregated into product-reaction contingency tables. Let ${n}_{11}$ denote the number of reports containing both a given product and a given reaction preferred term. The remaining cells of the 2×2 table were denoted by ${n}_{10}$, ${n}_{01}$, and ${n}_{00}$. From these counts, we computed the proportional reporting ratio (PRR), the reporting odds ratio (ROR), the 95% confidence interval of the ROR, and Pearson’s ${\chi }^{2}$ statistic. These measures remain among the most commonly used disproportionality metrics in pharmacovigilance signal detection (Bate et al. 1998; Bate and Evans 2009; Cutroneo et al. 2024; Evans et al. 2001; Fusaroli et al. 2024; van Puijenbroek et al. 2002). A product-reaction pair was classified as a candidate signal if it satisfied all of the following criteria:

$${n}_{11}\ge 3, \mathrm{P}\mathrm{R}\mathrm{R}\ge 2, {\chi }^{2}\ge 4$$

and the lower bound of the 95% confidence interval of the ROR was greater than 1. This rule combines standard frequency and disproportionality thresholds with an interval-based requirement for the ROR, thereby reducing emphasis on unstable low-count contrasts (Cutroneo et al. 2024; Evans et al. 2001; van Puijenbroek et al. 2002). A more conservative robust subset was then defined by the additional requirement ${n}_{11}\ge 10$, in order to prioritize pairs supported by higher reporting counts.

The resulting signals were interpreted strictly as prioritization candidates rather than as evidence of causality. As emphasized in the pharmacovigilance literature, disproportionality metrics are influenced by reporting biases, product utilization patterns, co-reporting structure, and database-specific features (Bate and Evans 2009; Cutroneo et al. 2024; Fusaroli et al. 2024; Harpaz et al. 2012). For this reason, the present analysis combined standard disproportionality screening with temporal persistence, overlap analysis, and sensitivity comparisons.

### Post-screening refinement and retained sets

After statistical screening, the quarterly robust signal sets underwent a two-step post-screening refinement procedure designed to improve interpretability in temporal comparisons. This step was rule-based and was applied consistently across quarters using pre-specified semantic and interpretive categories rather than ad hoc case-by-case judgment.

In the first step, product–reaction pairs were classified as excluded when the preferred term primarily reflected administrative, product-use-focused, or otherwise weakly informative content for clinical signal interpretation. These excluded categories included terms related to off-label use, medication errors, therapeutic misuse, product handling, and other operational or reporting-context descriptors. This yielded an intermediate refined set.

In the second step, pairs in the intermediate set were further classified into broader interpretive groups, including disease-related or indication-like terms, very general clinical terms, and product-use or device-related terms. Pairs not assigned to these categories constituted the final quarterly retained sets used for the main persistence and overlap analyses. Thus, the retained sets were defined as the subset of statistically screened pairs that remained after consistent exclusion or categorization of terms judged unlikely to support informative temporal comparison.

The operational categories used in this step were implemented through pre-specified rule-based term groupings applied consistently across quarters. Representative categories, operational descriptions, and example terms are provided in Supplementary Table S1. This procedure was intended to improve interpretability and temporal comparability of the retained signal sets and should be understood as a transparent rule-based prioritization step rather than as a substitute for formal validation.

This post-screening refinement was not intended as a substitute for statistical screening or as a form of external validation. Rather, it served as a pragmatic prioritization layer for constructing cleaner analytical sets for quarterly comparison. Accordingly, the retained sets should be interpreted as refined analytical objects for temporal signal characterization rather than as clinically validated signal lists.

### Annual persistence classes and overlap analysis

The quarterly retained sets were combined across the calendar year, and each product-reaction pair was classified according to the number of quarters in which it appeared. This yielded the persistence classes 1/4, 2/4, 3/4, and 4/4, with the 4/4 class representing the annual persistent core.

For the primary PS-clinical analysis, the annual union of retained pairs contained 82,565 distinct product-reaction pairs. Persistence classes were used to describe the distribution of quarterly detectability across the year and to identify the strongest members of the 4/4 core.

To assess continuity between quarters, we calculated pairwise Jaccard similarities and absolute intersections between retained quarterly sets. The Jaccard coefficient quantifies overlap relative to the union of two sets, whereas the absolute intersection reports the number of shared product-reaction pairs. These complementary summaries allowed us to compare quarterly overlap in both relative and absolute terms.

The same overlap analysis was performed under the broader PS + SS-clinical cohort. This sensitivity comparison was used to determine whether enlarging the cohort mainly changed the size of the retained signal space or also altered its temporal structure.

### Comparison of quarter-specific and fully persistent signals

To evaluate whether temporal persistence was associated with stronger statistical support, we compared retained pairs observed in only one quarter (1/4) with retained pairs observed in all four quarters (4/4). For each pair, quarterly disproportionality metrics were summarized by pair-level averages. We then compared the distributions of mean ${n}_{11}$, mean PRR and mean $X^2$ between the two persistence classes using the Mann–Whitney test.

This comparison was intended to determine whether repeated quarterly detection was primarily associated with larger support counts, stronger overall statistical evidence, greater relative disproportionality, or a combination of these features. Because the distributions of these metrics were markedly skewed, a rank-based nonparametric approach was preferred over normal-theory methods.

### Variability within the 4/4 core

Presence in all four quarters does not imply the same intensity of reporting across quarters. To describe within-core variability, we calculated for each 4/4 pair the ratio:

$$\frac{\mathrm{m}\mathrm{a}\mathrm{x}\left({n}_{11}\right)}{\mathrm{m}\mathrm{i}\mathrm{n}\left({n}_{11}\right)}$$

using the four quarterly ${n}_{11}$ values. We also computed the coefficient of variation (CV) across the same quarterly counts. Pairs satisfying

$$\frac{\mathrm{m}\mathrm{a}\mathrm{x}\left({n}_{11}\right)}{\mathrm{m}\mathrm{i}\mathrm{n}\left({n}_{11}\right)}\le 2$$

were classified as stable, whereas pairs satisfying

$$\frac{\mathrm{m}\mathrm{a}\mathrm{x}\left({n}_{11}\right)}{\mathrm{m}\mathrm{i}\mathrm{n}\left({n}_{11}\right)}\ge 5$$

were classified as highly variable. These thresholds were not intended as universal pharmacovigilance cutoffs, but rather as practical descriptive rules for separating a relatively balanced persistent core from a smaller subset with pronounced quarterly imbalance. This distinction was introduced to differentiate stable persistence from spike-driven persistence.

### Exploratory qualitative review of representative persistent signals

Finally, we conducted an exploratory qualitative review of representative members of the 4/4 core, focusing on the top 25 stable pairs and the top 25 highly variable pairs. Each reviewed pair received provisional annotations regarding clinical plausibility, whether the association appeared known or expected, whether it suggested possible reporting artifacts or contextual bias, and a final interpretive label (retain, caution, or likely artifact).

This review was intended only as a qualitative interpretive layer linking the statistical classification of persistent signals to their possible pharmacovigilance meaning. It should therefore be regarded as exploratory signal characterization rather than as formal validation.

#### Sensitivity analysis for cross-quarter repeated reports

Because FAERS quarterly public-use releases may contain repeated appearances of the same case across successive extracts, we conducted an additional sensitivity analysis to assess the possible effect of cross-quarter repeated reports on the temporal structure of the PS-clinical cohort. This analysis was performed at the case level prior to quarterly aggregation into product–reaction contingency tables, since the final quarterly robust and retained datasets were already aggregated by *medicinalproduct* and *reactionmeddrapt* and therefore no longer retained case identifiers.

Using the PS-clinical cohort, we first identified *safetyreportid* values appearing in more than one quarter. For each repeated report, we compared the set of associated drug–reaction pairs across quarters, where each pair was defined by the tuple (*safetyreportid, drugcharacterization, medicinalproduct, reactionmeddrapt*). Repeated reports whose pair sets were identical across quarterly appearances were treated as likely carryover duplicates. In a conservative sensitivity analysis, repeated pairs from such cases were deduplicated across quarters by retaining only the most recent observation according to *receiptdate*. Repeated reports with non-identical pair sets across quarters were not collapsed, since these were interpreted as potentially updated case versions rather than exact carryover duplicates. This analysis was intended to quantify the extent to which trivial cross-quarter carryover of identical repeated cases could inflate quarterly cohort size and pre-aggregation support counts.

## Results

### Quarterly summary under the primary PS-clinical cohort

Table 1 summarizes the quarterly results for the primary PS-clinical cohort. Across quarters, the number of evaluated product-reaction pairs ranged from 700,560 to 772,205. The number of robust signals with ${n}_{11}\ge 10$ ranged from 45,494 to 51,134. After post-screening refinement, the final retained sets ranged from 41,897 to 47,220 pairs. Q3 yielded the largest retained quarterly set, although the overall scale of the quarterly results remained broadly stable across the year.

**Table 1:** Quarterly summary of the PS-clinical signal detection workflow in FAERS, 2025

Quarter	Unique reports	Evaluated pairs	Robust signals	Refined	Excluded	Retained
Q1	223,449	717,631	49,587	47,933	2,515	45,418
Q2	220,734	700,560	45,494	43,923	2,026	41,897
Q3	252,805	772,205	51,134	49,403	2,183	47,220
Q4	216,833	723,982	49,247	47,611	2,082	45,529

### Annual persistence structure and the 4/4 core

Across the four quarterly retained sets, the annual union contained 82,565 unique product-reaction pairs. Of these, 34,739 (42.07%) appeared in only one quarter, 16,782 (20.33%) in two quarters, 12,415 (15.04%) in three quarters, and 18,629 (22.56%) in all four quarters (Table 2). Thus, although the largest fraction of retained signals was quarter-specific, nearly one quarter of the annual union formed a persistent 4/4 core.

**Table 2:** Annual persistence of retained PS-clinical signals across the four quarters of 2025

Persistence class	Quarters present	Number of pairs	Percent
1/4	1	34,739	42.07
2/4	2	16,782	20.33
3/4	3	12,415	15.04
4/4	4	18,629	22.56

Table 3 lists representative strong signals belonging to the 4/4 class. Some pairs, such as VEDOLIZUMAB-Haematochezia, showed persistent detection with relatively balanced quarterly support, whereas others, including OZEMPIC-Impaired gastric emptying and SPRAVATO-Dissociation, combined persistence with more pronounced temporal imbalance. Additional examples in the 4/4 class included VEDOLIZUMAB-Frequent bowel movements and NUPLAZID-Hallucination. Taken together, these examples illustrate that the persistent core contains both relatively stable and more unevenly expressed signals.

**Table 3:** Selected persistent 4/4 retained signals ranked by cumulative quarterly support

Medicinal product	Reaction PT	Q1	Q2	Q3	Q4	Sum	Mean
Vedolizumab	Haematochezia	679	713	718	722	2,832	708.00
Vedolizumab	Frequent bowel movements	593	619	603	659	2,474	618.50
Ozempic	Impaired gastric emptying	425	203	1422	423	2,473	618.25
Nuplazid	Hallucination	661	551	604	643	2,459	614.75
Ozempic	Nausea	405	294	834	508	2,041	510.25
Ozempic	Vomiting	336	283	694	491	1,804	451.00
Spravato	Dissociation	97	1104	265	260	1,726	431.50
Depo-provera	Meningioma	76	499	723	324	1,622	405.50
Tislelizumab	Myelosuppression	312	409	433	453	1,607	401.75
Cyclophosphamide	Febrile neutropenia	392	362	473	328	1,555	388.75

### Quarterly overlap of retained sets

**Fig. 1:**
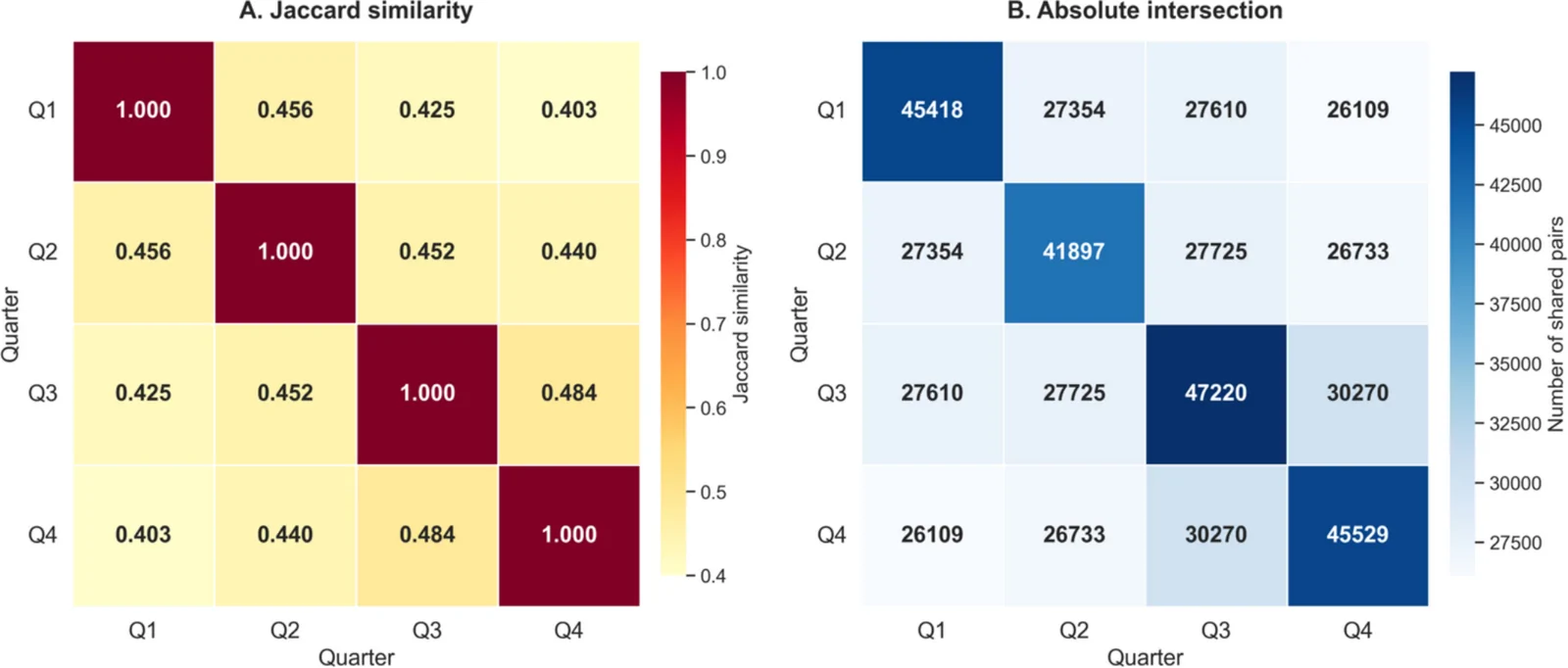
Pairwise overlap among quarterly retained PS-clinical signal sets in FAERS 2025. **A** shows the Jaccard similarity matrix for the retained quarterly sets. **B** shows the corresponding absolute intersection matrix, that is, the number of shared retained pairs between quarters. Together, the two panels indicate a moderate but structured degree of temporal continuity, with greater overlap between adjacent quarters and the strongest overlap between Q3 and Q4

Quarterly overlap analysis showed a moderate but structured degree of temporal continuity in the retained PS-clinical sets. Pairwise Jaccard similarities ranged from 0.4027 to 0.4845, indicating that quarterly retained sets were neither identical nor fully disjoint (Fig. 1). The highest overlap was observed between Q3 and Q4, whereas the lowest overlap was observed between Q1 and Q4.

A similar pattern was seen in absolute intersections. Q3 and Q4 shared 30,270 retained pairs, compared with 27,354 for Q1 and Q2 and 26,109 for Q1 and Q4. Overall, the two panels of Fig. 1 indicate that adjacent quarters tended to share a larger common core than quarters farther apart in time.

### Sensitivity to cohort definition: PS versus PS + SS

Expanding the cohort from PS-clinical to PS + SS-clinical substantially increased the size of the retained signal space in every quarter. The quarterly retained sets increased from 41,897–47,220 pairs under PS-clinical to 66,978–73,927 pairs under PS + SS-clinical. Relative increases ranged from 51.81% to 59.86%, depending on the quarter. Despite this expansion, overlap between the PS and PS + SS retained sets remained moderate and consistent, with quarterly Jaccard similarities ranging from 0.5166 to 0.5473.

**Fig. 2:**
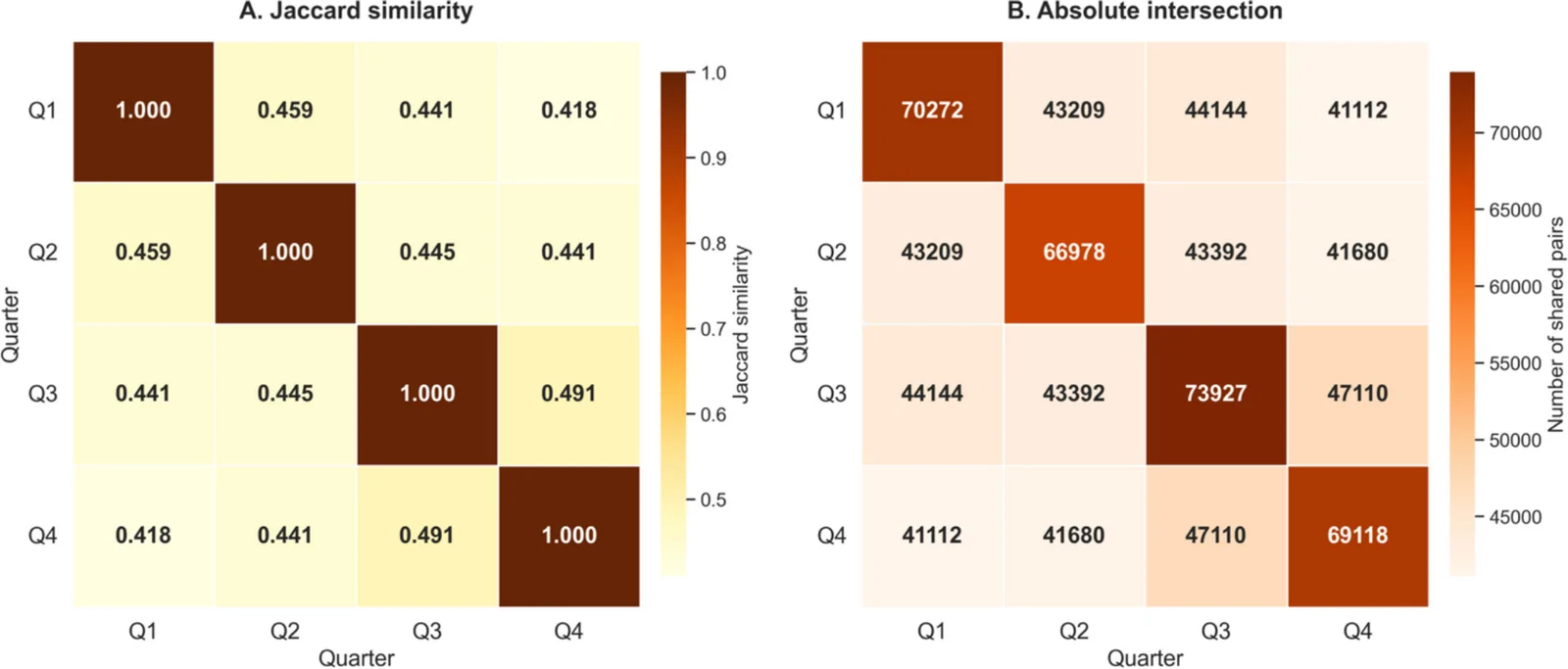
Pairwise overlap among quarterly retained PS + SS-clinical signal sets in FAERS 2025. **A** shows the Jaccard similarity matrix for the retained quarterly sets. **B** shows the corresponding absolute intersection matrix, that is, the number of shared retained pairs between quarters. Together, the two panels indicate moderate but structured temporal continuity in the broader PS + SS-clinical signal space, with the strongest overlap again observed between Q3 and Q4

**Fig. 3:**
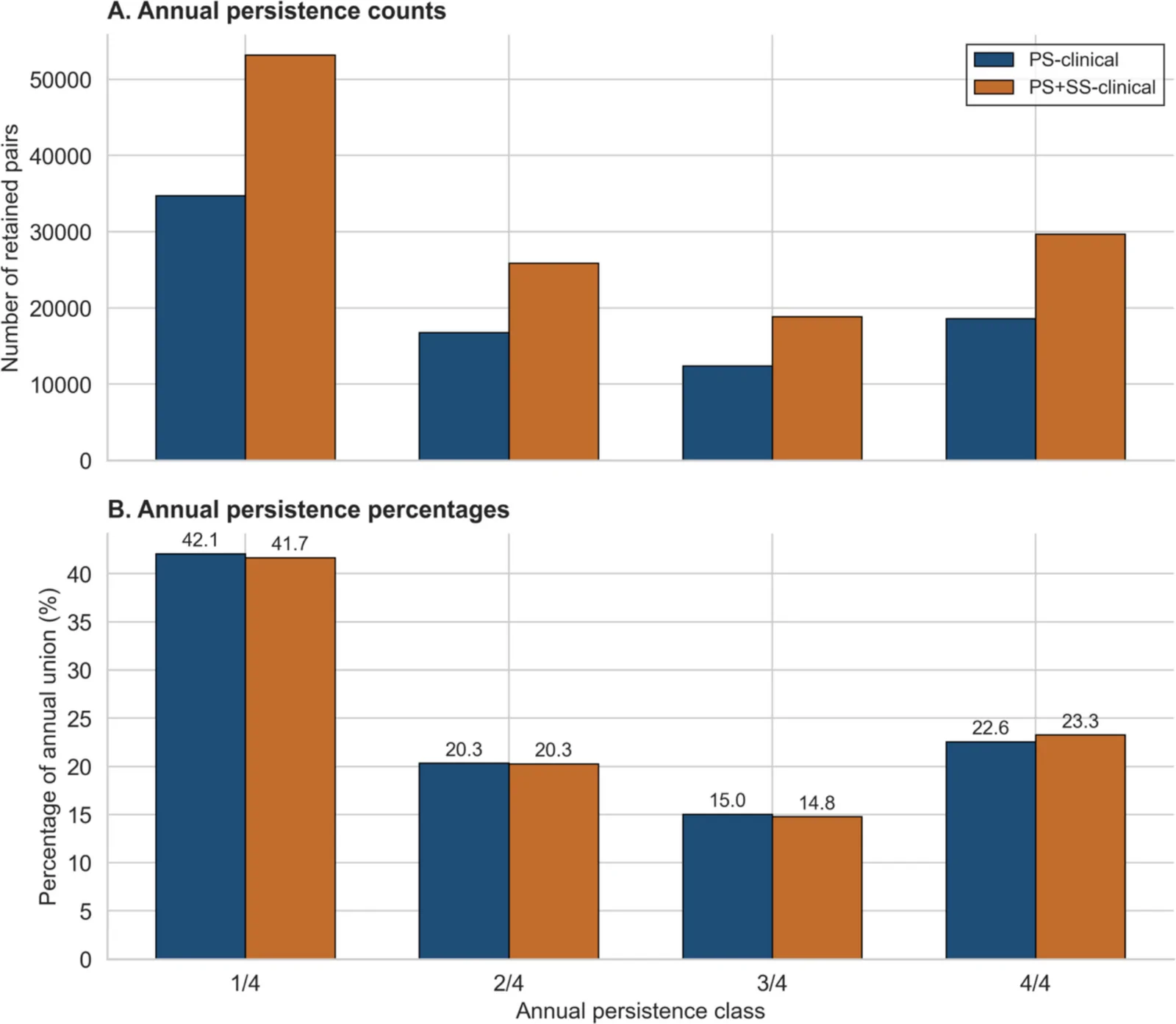
Comparison of annual persistence structure under the PS-clinical and PS + SS-clinical cohort definitions. **A** shows the number of retained pairs appearing in exactly one, two, three, or all four quarters of 2025. **B** shows the corresponding percentages relative to the annual union in each cohort. Although the PS + SS-clinical definition yields a larger retained signal space overall, the proportional persistence structure remains very similar to that observed under PS-clinical

Figure 2 shows that the broader PS + SS-clinical cohort preserved the same general overlap geometry observed under PS-clinical, with the strongest continuity again occurring between Q3 and Q4. At the annual level, the persistent 4/4 core increased from 18,629 pairs under PS-clinical to 29,699 under PS + SS-clinical. The intersection between both 4/4 cores was 15,959 pairs, corresponding to a Jaccard similarity of 0.493. Thus, broadening the cohort substantially changed the scale of the retained signal space but had a much smaller effect on its temporal structure. The proportional distribution across the 1/4 to 4/4 persistence classes remained highly similar under both cohort definitions (Fig. 3).

Table 4 summarizes the main annual comparison between the two cohort definitions. Likewise, Table 5 shows that absolute intersections increased under PS + SS for every quarterly pair, whereas the corresponding Jaccard similarities changed only modestly. These results indicate that inclusion of secondary suspect drugs expanded the retained signal space without materially altering its quarterly similarity structure.

**Table 4:** Annual sensitivity summary comparing PS-clinical and PS + SS-clinical cohort definitions

Metric	Cohort	Q1	Q2	Q3	Q4
Unique reports	PS-clinical	223,449	220,734	252,805	216,833
	PS + SS-clinical	223,995	221,407	253,547	217,566
Evaluated pairs	PS-clinical	717,631	700,560	772,205	723,982
	PS + SS-clinical	1,774,011	1,720,658	1,815,513	1,745,641
Robust ${n}_{11}\ge 10$	PS-clinical	49,587	45,494	51,134	49,247
	PS + SS-clinical	75,712	71,763	79,288	74,095
Retained	PS-clinical	45,418	41,897	47,220	45,529
	PS + SS-clinical	70,272	66,978	73,927	69,118

**Table 5:** Comparison of quarterly overlap between retained PS-clinical and PS + SS-clinical signal sets. For each pair of quarters, the table reports the Jaccard similarity, the absolute intersection size, and the corresponding differences between the two cohort definitions

Quarter pair	Jaccard (PS)	Jaccard (PS + SS)	Δ Jaccard	Intersection (PS)	Intersection (PS + SS)	Δ intersection
Q1–Q2	0.4562	0.4595	0.0033	27,354	43,209	15,855
Q1–Q3	0.4246	0.4412	0.0166	27,610	44,144	16,534
Q1–Q4	0.4027	0.4183	0.0156	26,109	41,112	15,003
Q2–Q3	0.4516	0.4450	− 0.0066	27,725	43,392	15,667
Q2–Q4	0.4405	0.4415	0.0010	26,733	41,680	14,947
Q3–Q4	0.4845	0.4911	0.0066	30,270	47,110	16,840

### Quarter-specific versus fully persistent signals

To assess whether annual persistence was associated with stronger statistical support, we compared retained pairs observed in only one quarter (1/4) with retained pairs observed in all four quarters (4/4). Because persistence classes were defined from quarterly retained signal sets that had already passed the screening criteria, this comparison should be interpreted as an internal descriptive contrast within the retained signal space. Fully persistent pairs had significantly higher average support counts than quarter-specific pairs, with a median mean ${n}_{11}$ of 23.75 compared to 12.00, respectively (Mann–Whitney U=99,826,101, $p<{10}^{-16}$). Similarly, 4/4 pairs showed stronger overall evidence based on the mean ${\chi }^{2}$, with medians of 157.19 against 90.65 (U=262,906,202, $p=4.35\times {10}^{-280}$).

**Fig. 4:**
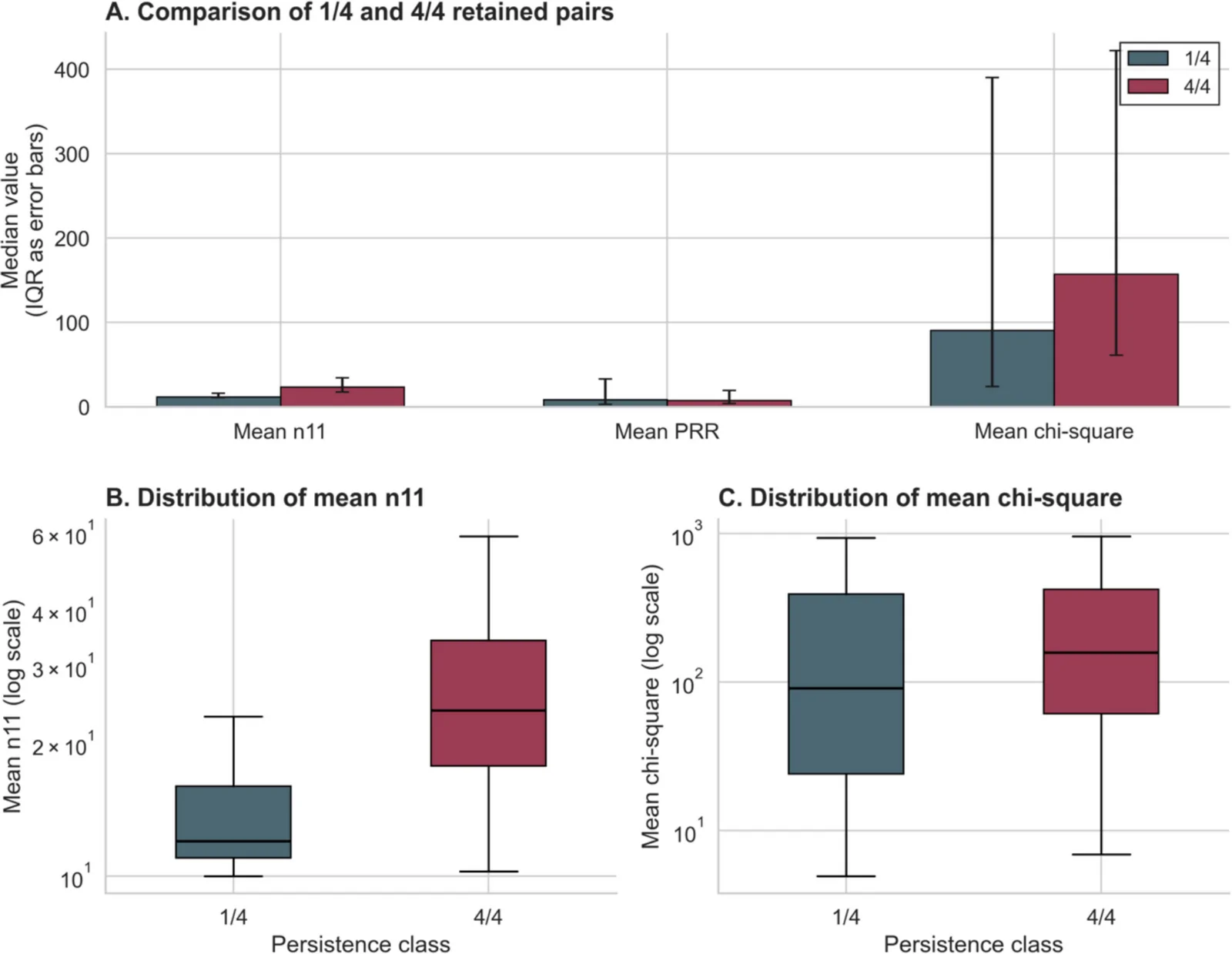
Comparison between quarter-specific (1/4) and fully persistent (4/4) retained pairs. **A** summarizes the median pair-level mean n11, PRR, and chi-square values, with interquartile ranges shown as error bars. **B** and **C** show the distributions of pair-level mean n11 and mean chi-square, respectively, on logarithmic scales. Fully persistent pairs were characterized by larger support counts and stronger chi-square evidence, whereas mean PRR did not differ materially between the two persistence classes

In contrast, mean PRR did not differ materially between the two persistence groups (median 7.93 in 4/4 vs 8.88 in 1/4; U=324,545,614, p=0.568). Figure 4 summarizes these results. Panel A presents median and interquartile range summaries of the three metrics, whereas Panels B and C display the distributions of pair-level mean ${n}_{11}$ and mean ${\chi }^{2}$ on logarithmic scales. Overall, annual persistence was associated with larger support counts and stronger statistical evidence within the retained signal space, but not with consistently larger PRR values. However, these differences should be interpreted descriptively, since the comparison groups were defined from the same quarterly screening framework.

### Stable versus highly variable signals within the 4/4 core

The presence in all four quarters did not imply uniform reporting intensity. Within the annual 4/4 core, most retained pairs showed relatively balanced quarterly support, whereas only a small subset exhibited marked temporal imbalance. Using the ratio $\mathrm{m}\mathrm{a}\mathrm{x}\left({n}_{11}\right)/\mathrm{m}\mathrm{i}\mathrm{n}\left({n}_{11}\right),$ 10,991 pairs were classified as stable (≤2), while 150 were classified as highly variable (≥5).

Despite their smaller number, highly variable pairs had substantially larger support. Their median total ${n}_{11}$ was 171.0 compared to 86.0 in the stable group, and their median mean ${n}_{11}$ was 42.75, whereas the stable group had 21.50 (Mann–Whitney U=241,526.5, $p=3.47\times {10}^{-50}$ for both comparisons). As expected, highly variable pairs also showed much greater variation over time, with a median $\mathrm{m}\mathrm{a}\mathrm{x}/\mathrm{m}\mathrm{i}\mathrm{n}$ ratio of 5.92 compared to 1.60 and a median coefficient of variation of 0.755 versus 0.209 ($p<{10}^{-90}$ for both).

**Fig. 5:**
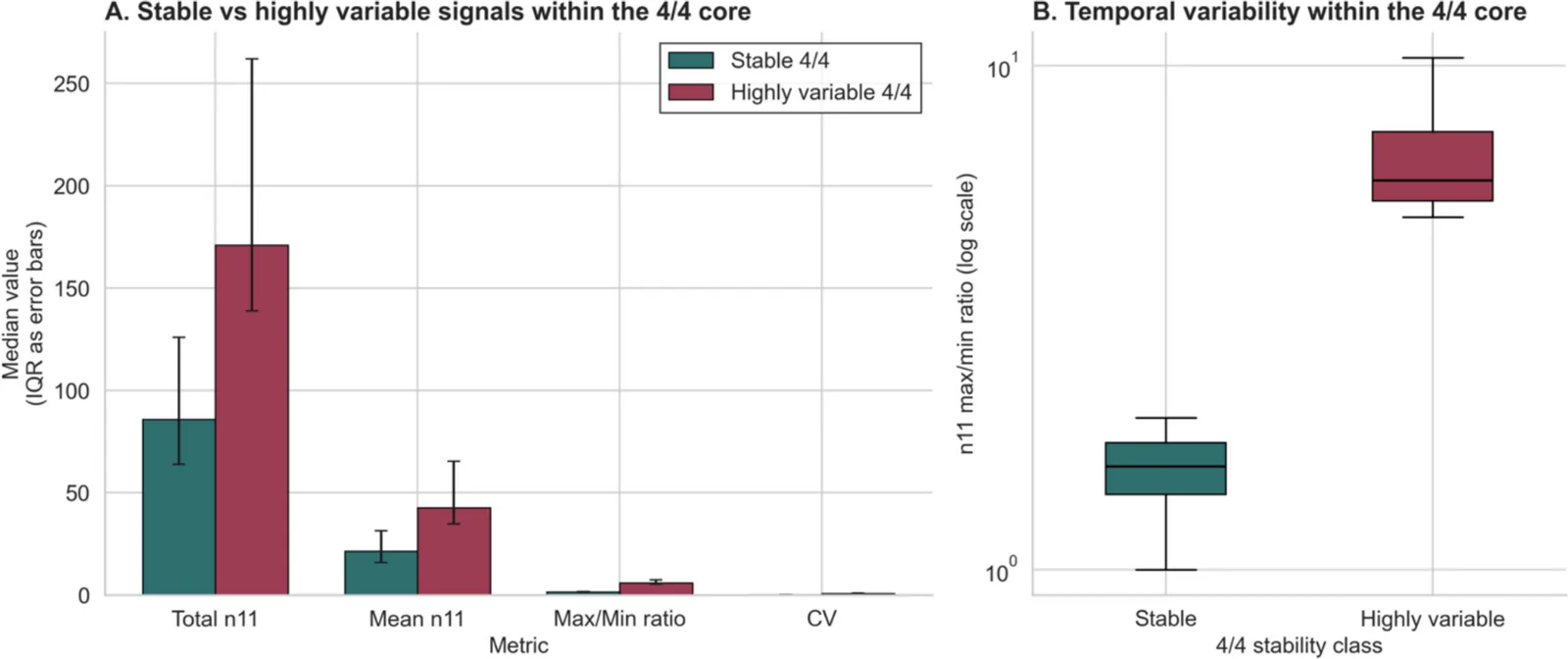
Comparison between stable and highly variable retained pairs within the annual 4/4 core. **A** shows median total n11, mean n11, max/min ratio, and coefficient of variation (CV), with interquartile ranges represented as error bars. **B** shows the distribution of the quarterly support ratio max(n11)/min(n11) on a logarithmic scale. Highly variable pairs constitute a small subset of the 4/4 core but exhibit greater support and markedly stronger temporal dispersion than stable pairs

Figure 5 illustrates this contrast. Panel A summarizes differences in overall support and temporal dispersion, whereas Panel B displays the distribution of the quarterly support ratio on a logarithmic scale. These results indicate that the 4/4 core was dominated by relatively stable associations, with only a small subset showing strong but uneven quarterly intensity.

### Exploratory qualitative review of representative persistent signals

To complement the quantitative classification of the 4/4 core, we conducted an exploratory qualitative review of the top 25 stable and top 25 highly variable 4/4 pairs. Under this provisional annotation, highly variable pairs did not appear systematically less plausible than stable pairs. In the reviewed sample, 72% of highly variable pairs were categorized as clinically plausible, compared with 60% of stable pairs. Likewise, 64% of highly variable pairs were annotated as known or expected, compared with 56% of stable pairs. Provisional retain labels were assigned to 40% of highly variable pairs and 28% of stable pairs, whereas likely artifact labels were assigned to 20% and 28%, respectively.

**Fig. 6:**
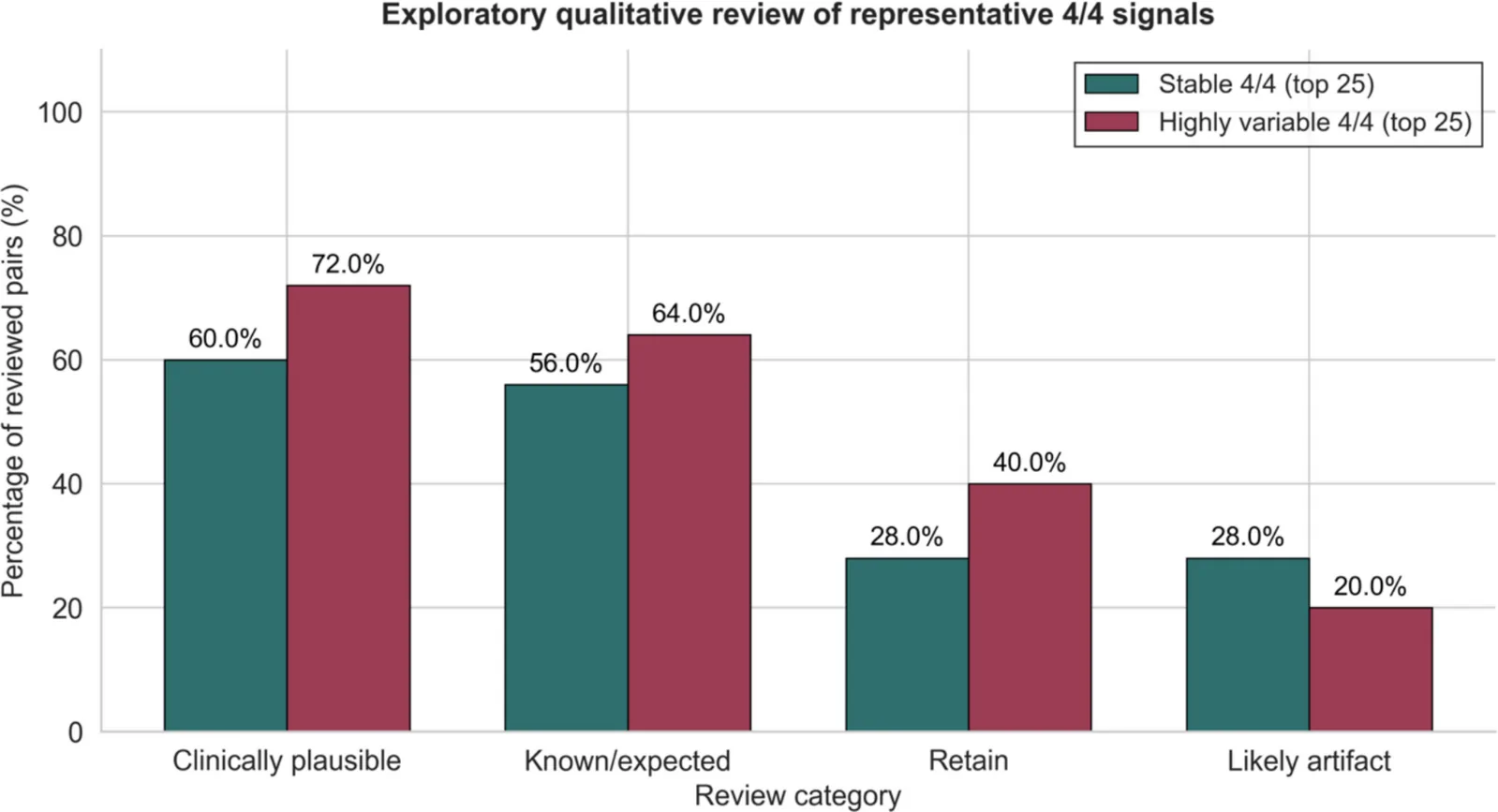
Exploratory qualitative review of representative 4/4 retained pairs. The figure compares the top 25 stable pairs and the top 25 highly variable pairs according to the percentage provisionally classified as clinically plausible, known or expected, retained after review, and likely artifact. The comparison suggests that temporal variability within the 4/4 core does not necessarily imply lower plausibility

Figure 6 summarizes these exploratory results and highlights an important interpretive point: temporal variability alone did not imply lower plausibility. Some highly variable persistent pairs may still represent clinically meaningful associations despite marked changes in reporting intensity over time. Stability and plausibility should therefore be regarded as related, but distinct, dimensions.

### Sensitivity to cross-quarter repeated reports

To assess the potential influence of repeated case appearances across quarterly FAERS releases, we performed a conservative sensitivity analysis in the PS-clinical cohort at the case level prior to quarterly aggregation. A total of 85,112 *safetyreportid* values appeared in more than one quarter. Among these repeated reports, 47,839 had identical drug–reaction pair sets across quarterly appearances and were therefore treated as likely carryover duplicates, whereas the remaining repeated reports showed non-identical pair sets and were retained as potentially updated case versions.

Removing repeated pairs from the identical repeated cases eliminated 880,638 rows, corresponding to 3.70% of the PS-clinical cohort. The largest reductions occurred in Q1 and Q2, smaller reductions in Q3, and no reduction in Q4. At the quarterly cohort level, the numbers of unique *medicinalproduct–reactionmeddrapt* pairs decreased from 717,631 to 677,235 in Q1, from 700,560 to 673,567 in Q2, and from 772,205 to 763,138 in Q3, whereas Q4 remained unchanged. The corresponding relative decreases were 5.63%, 3.85%, 1.17%, and 0.00%, respectively.

When quarterly pair-level support was reconstructed as the number of unique *safetyreportid* values per (*medicinalproduct, reactionmeddrapt*) combination, the number of pairs with *n*_*11*_ ≥ 10 decreased from 103,688 to 97,849 in Q1, from 95,209 to 90,978 in Q2, and from 112,881 to 110,755 in Q3, again with no change in Q4. Thus, cross-quarter carryover of identical repeated cases does inflate pre-aggregation support counts to some extent, particularly in earlier quarters, but the magnitude of this effect was moderate under the conservative deduplication rule.

Because the final quarterly robust and retained signal sets used in the manuscript were produced after aggregation and editorial refinement, this sensitivity analysis does not replace the published retained-set results directly. Rather, it provides a robustness check showing that repeated cross-quarter carryover contributes to some inflation of temporal summaries, especially in earlier quarters, while not by itself fully accounting for the broader temporal structure observed in the primary analysis.

## Discussion

The present findings indicate that quarterly FAERS signal detection can be interpreted as a temporally structured process rather than as a sequence of isolated disproportionality snapshots. Under the primary PS-clinical cohort, the retained quarterly sets were large but broadly stable in scale, yielding both a substantial annual union and a persistent 4/4 core. At the same time, overlap analyses showed moderate continuity rather than complete stability or random turnover, with adjacent quarters sharing more retained pairs than more distant ones. Taken together, these results suggest that quarterly FAERS analyses are better understood as related temporal signal sets rather than as independent quarterly outputs (Bate and Evans 2009; Cutroneo et al. 2024; Fusaroli et al. 2024; Huang et al. 2014).

The comparison between quarter-specific (1/4) and fully persistent (4/4) signals further supports this interpretation. Fully persistent pairs showed larger support counts and stronger overall ${\chi }^{2}$ evidence, whereas mean PRR did not differ materially between persistence classes. This pattern suggests that quarterly persistence is more closely related to repeated empirical support and cumulative statistical stability than to systematically larger relative disproportionality. In this sense, persistence may add information that is complementary to, rather than redundant with, standard disproportionality measures. Repeated quarterly detectability appears to identify pairs that remain analytically reproducible across releases even when their relative disproportionality is not consistently higher (Candore et al. 2015; Jiao et al. 2024; Khouri et al. 2021).

An important caveat is that persistence class membership was defined from quarterly retained signal sets that had already passed the screening criteria, including support-based thresholds. Accordingly, the observed differences between 1/4 and 4/4 pairs in *n*_*11*_ and to some extent in related disproportionality summaries are partly conditioned by the same screening process used to construct the comparison groups. For this reason, these contrasts should be interpreted primarily as descriptive internal differences within the retained signal space rather than as fully independent evidence that persistent pairs are intrinsically superior prioritization targets.

Previous methodological work in pharmacovigilance has mainly emphasized variation in signal detection performance across disproportionality algorithms, databases, and analytical settings (Candore et al. 2015; Khouri et al. 2021; Vogel et al. 2020), together with broader guidance on the interpretation and practical use of disproportionality analyses (Cutroneo et al. 2024; Fusaroli et al. 2024). In contrast, less attention has been given to repeated detectability across successive quarterly public releases as an additional prioritization dimension. From this perspective, the present study does not replace conventional disproportionality screening, but extends it by examining quarterly persistence, overlap structure, and within-core variability as complementary descriptors of the retained signal space.

The sensitivity analysis under the broader PS + SS-clinical cohort provides additional support for the robustness of the main temporal findings. Inclusion of secondary suspect drugs markedly expanded the retained signal space and enlarged the annual 4/4 core, but it had a much smaller effect on the overall persistence profile and overlap geometry. The strongest continuity again occurred between adjacent quarters, especially Q3 and Q4. This suggests that the temporal organization of the retained signal space is not merely an artifact of the stricter primary-suspect definition. More broadly, these findings support the view that sensitivity analyses in pharmacovigilance are useful not only for quantifying scale changes, but also for assessing whether higher-level temporal structure remains stable under alternative cohort definitions (Candore et al. 2015; Khouri et al. 2021; Coste et al. 2023; Vogel et al. 2020).

An additional methodological sensitivity analysis addressed the potential effect of cross-quarter repeated reports in the PS-clinical cohort. Repeated *safetyreportid* values were observed across quarterly releases, and a subset of these showed identical drug–reaction pair sets across quarters, consistent with likely carryover duplicates. Under a conservative deduplication rule retaining only the most recent observation for such identical repeated cases, the largest effects were observed in Q1 and Q2, smaller effects in Q3, and no effect in Q4. At the same time, the magnitude of change in reconstructed quarterly pair-level support counts remained moderate. This suggests that repeated case carryover can inflate temporal summaries to some extent, particularly in earlier quarters, but does not by itself fully account for the broader temporal structure observed in the primary analysis. Accordingly, persistence and overlap summaries should be interpreted with additional caution, especially for lower persistence classes and earlier quarters.

The distinction between stable and highly variable members of the 4/4 core adds an additional descriptive layer. Persistence across all four quarters did not imply uniform support over time. Most persistent pairs were relatively balanced across quarters, whereas a much smaller subset combined persistence with pronounced temporal imbalance. Such highly variable persistent pairs may reflect changes in exposure, reporting practices, product utilization, external attention, or broader shifts in reporting behavior. They should therefore not be dismissed automatically, but neither should they be interpreted in the same way as more stable persistent pairs. From a practical standpoint, distinguishing stable persistence from spike-driven persistence may help refine pharmacovigilance prioritization within the persistent core (Cutroneo et al. 2024; Fusaroli et al. 2024; Khouri et al. 2021).

The exploratory qualitative review provides a further cautionary perspective. In the reviewed subset, highly variable persistent pairs did not appear systematically less plausible than stable persistent pairs. This suggests that temporal imbalance alone should not be taken as evidence of lower relevance. At the same time, the qualitative review also highlights the need for caution when interpreting indication-like preferred terms, non-specific clinical language, and context-sensitive reporting categories. Accordingly, this qualitative component should be interpreted as exploratory signal characterization rather than validation, consistent with current methodological guidance distinguishing statistical detection, clinical interpretation, and causal assessment (Cutroneo et al. 2024; Fusaroli et al. 2024).

An important qualification is that the present study evaluates persistence only as an internal property of retained FAERS signal sets. Accordingly, our results support quarterly persistence as an interpretable analytical and prioritization-oriented dimension within FAERS-based signal detection, but they do not establish that persistent pairs are more likely to correspond to externally confirmed or subsequently validated safety signals, demonstrating that this stronger form of prioritization utility would require comparison against external reference standards, such as regulatory communications, labeled signal sets, drug label changes, or other downstream validation sources. From this perspective, the present work should be understood as a methodological and descriptive framework rather than as an externally validated ranking system.

Overall, the present results suggest that quarterly persistence may be useful as an additional internal analytical and prioritization-oriented dimension in FAERS-based signal detection. Rather than replacing disproportionality metrics, persistence may complement them by helping distinguish quarter-specific associations, reproducible annual cores, and persistent but unevenly expressed signals. From a methodological perspective, this approach extends conventional snapshot-based pharmacovigilance workflows toward a more explicitly temporal description of retained signal spaces. From a practical perspective, it offers an interpretable framework for organizing large quarterly signal sets into reproducible temporal categories that may support downstream review and prioritization.

## Conclusions

This study developed a quarterly FAERS-based signal detection workflow focused on temporal persistence, overlap structure, cohort sensitivity, and within-core variability. Starting from the 12 public-use XML files released in 2025, a chunked parsing strategy was used to generate report-level drug-reaction pairs for quarterly disproportionality screening and annual synthesis. Under the primary PS-clinical cohort, the annual union of retained signals comprised 82,565 distinct product-reaction pairs, of which 18,629 were detected in all four quarters.

Several main conclusions emerge from these results. First, the retained quarterly sets showed moderate but consistent temporal overlap, with adjacent quarters sharing a larger common core than more distant quarters. This suggests that the retained signal space changed gradually over the year rather than through abrupt quarter-to-quarter turnover. Second, fully persistent 4/4 pairs showed larger support counts and stronger ${\chi }^{2}$ evidence than quarter-specific 1/4 pairs, whereas mean PRR did not differ materially between the two groups. Thus, annual persistence appears in this analysis to reflect repeated empirical support and cumulative stability more than systematically larger relative disproportionality. Third, the persistent 4/4 core was heterogeneous: most pairs were relatively stable across quarters, whereas a much smaller subset showed pronounced temporal imbalance. Fourth, expansion of the cohort from PS to PS + SS substantially enlarged the retained signal space and the annual 4/4 core, while leaving the main temporal structure largely unchanged. Finally, the exploratory qualitative review suggested that temporal variability alone did not imply lower plausibility, since some highly variable persistent pairs remained potentially meaningful from a pharmacovigilance perspective.

Taken together, these findings support quarterly persistence as an interpretable additional dimension for internal FAERS-based signal characterization and prioritization. Rather than replacing disproportionality analysis, persistence may complement it by highlighting reproducibility, continuity, and temporal structure within retained FAERS signal sets. External validation against independent pharmacovigilance reference standards remains an important direction for future work.

## Limitations and outlook

This study has several limitations. First, the analysis is based on spontaneous reporting data and therefore inherits the well-known limitations of FAERS, including under-reporting, stimulated reporting, variable data quality, missing exposure denominators, and the inability to support causal inference directly. Second, although the post-screening refinement was based on explicit semantic and interpretive categories applied consistently across quarters, it remained a prioritization-oriented analytical step rather than a form of formal external validation. Likewise, the qualitative review was intended only for interpretive support. In addition, although the refinement procedure was rule-based, we did not formally evaluate inter-rater reliability or benchmark alternative refinement schemes. Therefore, some dependence on the chosen operational categorization remains, and future work should examine the robustness of the retained signal sets under alternative refinement decisions. Third, the present study did not evaluate whether persistent pairs were more likely than quarter-specific pairs to correspond to externally confirmed or subsequently validated safety signals. Therefore, the prioritization value of persistence was assessed only internally within FAERS and should not be interpreted as externally validated signal ranking performance. Fourth, comparisons between persistence classes on screening-related metrics, particularly *n*_11_, are partly conditioned by the same quarterly screening procedure used to define the retained signal sets. Therefore, differences between quarter-specific and fully persistent pairs in support-related summaries should be interpreted as internal descriptive contrasts rather than as fully non-circular evidence of prioritization utility. Fifth, although we performed a conservative sensitivity analysis for cross-quarter repeated reports, the final retained quarterly datasets used in the main manuscript were already aggregated by product–reaction pair and therefore did not allow exact case-level deduplication at the final analytical stage. The additional sensitivity analysis showed that repeated case carryover can inflate pre-aggregation support counts to some extent, particularly in earlier quarters, but a fully integrated reconstruction of the retained-set pipeline under case-level cross-quarter deduplication was beyond the scope of the present study. Sixth, the classification of stable versus highly variable members of the 4/4 core was based on simple summaries of quarterly support and should therefore be interpreted as a practical descriptive device rather than as a substitute for more detailed exposure-adjusted or longitudinal modeling. Seventh, the temporal dimension examined here was defined by repeated detectability across quarterly public releases rather than by an explicit model of reporting-time dynamics. Accordingly, persistence should be interpreted as repeated analytical detectability across releases, not as direct evidence of long-term causal stability.

Several extensions of this framework appear feasible. One natural next step would be to incorporate product classes or grouped reaction categories in order to assess whether persistence differs across therapeutic or clinical domains. Another would be to compare FAERS-based persistence with external knowledge sources, labeled signal sets, regulatory communications, or drug label changes. A further priority would be to reconstruct the full retained-set workflow under case-level cross-quarter deduplication so that quarterly overlap, persistence classes, and retained signal sets can be evaluated under a unified duplicate-handling framework. More specific temporal or hierarchical models could also help distinguish stable persistence from persistence driven by notoriety, changing exposure, or other reporting-related dynamics. Future work could also formalize post-screening categorization further through externally curated terminology rules or semi-automated classification procedures and evaluate the stability of retained signal sets under alternative refinement decisions. Finally, it would be useful to evaluate whether repeated quarterly detection improves downstream signal prioritization relative to conventional single-snapshot screening. Such developments may help clarify which persistent signals are most informative for pharmacovigilance practice and which are better understood as transient reporting patterns.

Supplementary information.

## Supplementary Information

Below is the link to the electronic supplementary material.

ESM 1 (DOCX.14.6 KB)

## Data Availability

The raw data used in this study are publicly available through the U.S. Food and Drug Administration Adverse Event Reporting System (FAERS) quarterly public-use XML releases for 2025. Derived analytical tables, processed summary files, and code supporting the main results are available from the corresponding author upon reasonable request.

## Declarations

### Generative AI and related technologies

The authors take full responsibility for the content of this manuscript, including the accuracy, integrity, and originality of the text, analyses, and interpretations presented. No paper mill or undisclosed third-party writing service was used in the preparation of this work.
