# Supplementary material for: Quarterly persistence as a signal prioritization framework in FAERS: an analysis of the 2025 public-use releases
Source: Naunyn Schmiedebergs Arch Pharmacol. 2026 Jun 8;399(11):18199–213. doi: 10.1007/s00210-026-05505-4 (PMC13503449; doi:10.1007/s00210-026-05505-4)
Supplement: Supplementary file 1 [file 210_2026_5505_MOESM1_ESM.docx]

Supplementary Table S1. Operational categories used in the post-screening refinement procedure

| **Category** | **Operational description** | **Representative terms or term types** | **Action taken** |
| --- | --- | --- | --- |
| Administrative / reporting-context terms | Preferred terms primarily reflecting administrative handling, reporting workflow, or documentation context rather than a clinically interpretable adverse event pattern. | *Off label use*, *medication error*, *incorrect dose administered*, reporting-context descriptors, documentation-related terms | Excluded at step 1 |
| Product-use-focused terms | Preferred terms mainly describing product use, misuse, non-standard use, or operational aspects of administration rather than a clinically informative adverse event signal. | *Therapeutic misuse*, product use in unapproved indication, dose omission or administration-related issue terms | Excluded at step 1 |
| Product handling / operational terms | Terms focused on handling, storage, dispensing, preparation, or other operational product-process descriptors with limited value for temporal signal interpretation. | Product handling terms, preparation/administration process descriptors, operational product categories | Excluded at step 1 |
| Disease-related or indication-like terms | Preferred terms more suggestive of the underlying condition, treatment indication, or disease label than of a distinct adverse event suitable for temporal comparison. | Disease names, indication-like terms, treatment-target conditions, terms closely aligned with the therapeutic indication | Categorized at step 2; not retained in final analytical set |
| Very general clinical terms | Terms that are clinically broad, nonspecific, or insufficiently informative for stable temporal comparison across quarters. | Highly generic descriptors such as *illness*, nonspecific pain-related terms, broad constitutional or poorly resolved symptom terms | Categorized at step 2; not retained in final analytical set |
| Product-use or device-related terms | Terms referring mainly to use context, device/process interaction, or product-associated procedural language rather than a clinically interpretable adverse event pattern. | Device-related descriptors, administration-context terms, use-related procedural terms | Categorized at step 2; not retained in final analytical set |
| Retained clinically interpretable terms | Preferred terms not assigned to the exclusion or categorization groups above and considered sufficiently specific for temporal comparison after statistical screening. | Reaction preferred terms with clearer adverse-event interpretation, including clinically recognizable event patterns | Retained for persistence and overlap analyses |

**Note:** The post-screening refinement was implemented as a rule-based semantic filtering step applied consistently across quarters. The categories and examples shown here summarize the operational codebook used in the analysis. The examples are representative and not intended to be exhaustive.
